# Anxiety and physical impairment in patients with central vestibular disorders

**DOI:** 10.1007/s00415-023-11871-3

**Published:** 2023-08-08

**Authors:** Lena Padovan, Sandra Becker-Bense, Virginia L. Flanagin, Ralf Strobl, Karina Limburg, Claas Lahmann, Julian Decker, Marianne Dieterich

**Affiliations:** 1 German Center for Vertigo and Balance Disorders, University Hospital, Ludwig-Maximilians-Universität München, Marchioninistr. 15, 81377 Munich, Germany; 2 Institute for Medical Information Processing Biometry and Epidemiology (IBE), Ludwig-Maximilians-Universität München, Munich, Germany; 3 Clinic for Conservative Orthopaedics, Manual Medicine and Pain Medicine, Sana Klinik München, Munich, Germany; 4 Department of Psychosomatic Medicine and Psychotherapy, University Medical Center, Albert-Ludwigs-University Freiburg, Freiburg, Germany; 5 Department of Neurology, Schön Klinik Bad Aibling, Bad Aibling, Germany; 6 Department of Neurology, University Hospital, Ludwig-Maximilians-Universität München, Munich, Germany; 7 Munich Cluster for Systems Neurology (SyNergy), Munich, Germany

**Keywords:** Vestibular, Anxiety, Activity, Central, Vertigo-related anxiety, Vertigo handicap questionnaire

## Abstract

**Background:**

There is increasing evidence for close interrelations between vestibular and emotional brain networks. A study in patients with bilateral peripheral vestibulopathy (BVP) showed relatively low vertigo-related anxiety (VRA), despite high physical impairment. The current working hypothesis proposes the integrity of the peripheral vestibular system as a prerequisite for development of VRA. Here we contribute by evaluating VRA and vestibular-related handicap in central vestibular disorders.

**Methods:**

Of 6396 patients presenting in a tertiary vertigo centre, 306 were identified with four clear central vestibular disorders: pure cerebellar ocular motor disorder (COD; 61), cerebellar ataxia (CA; 63), atypical parkinsonian syndromes (APS; 28), vestibular migraine (VM; 154). Their results of the Vertigo Handicap Questionnaire (VHQ), with its subscales for anxiety and handicapped activity, were compared to those of 65 BVP patients. Postural instability was measured on a force-plate. Multivariate linear regression was used to adjust for patient demographics.

**Results:**

Patients with chronic central vestibular disorders (COD, CA, APS) had relatively low VRA levels comparable to those in BVP, independent of increased handicapped activity or postural instability. Only VM patients showed significantly higher VRA, although their activity impairment and postural instability were lowest. No significant differences within chronic central vestibular disorders were found for VRA and subjective activity impairment.

**Conclusions:**

Subjective and objective vestibular-related impairment are not necessarily correlated with vestibular-related anxiety in central vestibular disorders. Our findings rather support the hypothesis that, in addition to an intact peripheral, an intact central vestibular system could also serve as a prerequisite to develop specific VRA.

**Supplementary Information:**

The online version contains supplementary material available at 10.1007/s00415-023-11871-3.

## Introduction

Vertigo and dizziness are common complaints of patients at all ages with a lifetime prevalence of around 15–35% in the general population [1]. They are a multisensory and sensorimotor syndrome with perceptual, postural, ocular motor and autonomic manifestations that is usually caused by a mismatch of information between vestibular, visual, and somatosensory systems. The relevant structures of the vestibular system run bilaterally from the inner ear, via the vestibular nerve, brainstem and cerebellum upward to temporo-parietal multisensory cortical network areas.

In addition to marked functional impairment, vertigo and dizziness significantly impact psychological health [2]. The various vertigo syndromes differed significantly in terms of psychological distress in the acute phase and even more so at repeated follow-up examinations over a year [3–6], irrespective of vestibular function [7]. The assessment of a manifest psychiatric comorbidity revealed a significantly higher prevalence in patients presenting to specialised vertigo centres (40–60%), than that expressed in the general population (20–30%) [3, 6, 8]. Again, large differences were seen between different vestibular syndromes, e.g. manifest anxiety disorders were found in almost 50% of patients with vestibular migraine (VM), but in only in 24% with bilateral loss of peripheral vestibular function (bilateral vestibulopathy; BVP) [3–6].

While general anxiety disorders are highly individual and complex in genesis [9], in the past the question arose of how the symptom of vertigo and dizziness itself affects symptom-specific anxiety, independent of a manifest psychiatric disorder. This includes vertigo-related phobic avoidance, anxious beliefs and also social anxiety. For this purpose, various questionnaires have already been evaluated in previous studies [10], whereby the Vertigo Handicap Questionnaire (VHQ) with its subgroups for anxiety and activity-related impairment appeared to be a promising tool [11]. A study by Decker and colleagues assessing vertigo-related anxiety (VRA) in 7083 patients with the key symptoms of vertigo or dizziness by using the VHQ subscales found significantly lower scores in BVP patients despite their well-known high activity impairment and increased risk of falling compared to patients with preserved peripheral-vestibular function, e.g., VM or functional dizziness [12–14]. From this, the current hypothesis was derived that an intact peripheral vestibular system is a prerequisite for the development of VRA thereby substantiating the close linkage between the vestibular and anxiety systems [15].

There are several studies demonstrating the structural and functional relationships between the vestibular system and emotional, cognitive and visceral functions in humans and other animals [16–20] comprising reciprocal connections of thalamocortical and limbic pathways [16, 20], connections with the cerebellum [20, 21] as well as noradrenergic and serotonergic projections [21, 22]. In a recent MRI meta-analysis, the areas of overlap between the vestibular system and the anxiety system were demonstrated in the upper brainstem and cortex [23].

While the influence of bilateral peripheral vestibular hypofunction on anxiety and especially on VRA is just starting to be understood, the influence of persistent central vestibular disorders on emotional processing is still largely unknown. A study on psychiatric comorbidity among patients with vestibular disorders reported generally increased comorbidity rates in “central vertigo” by about 42.1%, but the prevalence of anxiety disorders was comparably low to that of BVP (18.4% vs.17.8%) [6]. However, the number of patients with “central vertigo” enrolled was relatively small, and their aetiology too heterogeneous to allow more precise statements. The aim of the current study therefore was to specifically evaluate vertigo and dizziness related anxiety and handicapped activity in different types of central vestibular disorders.

## Methods

### Study collective and neuro-otological examination

6396 patients, who presented to the German Center for Vertigo and Balance Disorders (DSGZ, University Hospital, LMU Munich, Germany) between 2015 and 2018 with the chief complaints of vertigo, dizziness, or balance disorders, were screened retrospectively to identify patients with central vestibular disorders. Inclusion criteria were a minimum age of 18 years and sufficient language skills. Patients with multifactorial central pathology were excluded from the analyses.

All patients underwent a detailed medical history taking and a standardised clinical neurological examination, especially including precise sensory testing and positioning manoeuvres. Furthermore, all patients underwent a specialized neuro-otological and neuro-orthoptic examination including a visual acuity test (Snellen chart), measurements of the subjective visual vertical (SVV, normal range 0° ± 2.5°) and fundus photography to test for acute vestibular tone imbalance in the roll plane [24]. Video-oculography (EyeSeeCam^®^, Interacoustics, Denmark) was applied during the horizontal head impulse test to analyse the vestibulo-ocular reflex (VOR) in the high-frequency range and bithermal caloric testing of the horizontal semicircular canal function in the low-frequency range in a standardised manner [25, 26]. If useful additional audiometry, vestibular evoked myogenic potentials, eye movement recording, gait analysis, or posturography were performed [25].

A total of 152 patients with specific chronic central vestibular disorders were identified from the retrospective cohort and classified into three subgroups:

1. “Pure” cerebellar ocular motor disorders (COD) such as downbeat nystagmus syndrome without other signs of cerebellar dysfunction (n = 61) [27].
2. Cerebellar ataxia syndromes (CA) in the presence of cerebellar ocular motor disorders and further signs of cerebellar dysfunction, e.g., gait, truncal or limb ataxia, dysarthria, tremor or dysdiadochokinesia (n = 63; 30 idiopathic late-onset cerebellar ataxia, 10 cerebellar ataxia with neuropathy and bilateral vestibular areflexia syndromes (CANVAS); 7 spinocerebellar ataxia, 3 episodic ataxia type II, 4 other hereditary ataxia; 2 autoimmune, 1 metabolic, 4 post ischemia, 2 post intracranial haemorrhages) [28].
3. Atypical Parkinsonian Syndromes (APS, n = 28) caused by multiple system atrophy of cerebellar (MSA-C, n = 15) or parkinsonian type (MSA-P, n = 4), or progressive supranuclear palsy (PSP, n = 9) [29, 30].

154 patients with vestibular migraine (VM) representing an episodic central vestibular syndrome, and 65 patients with BVP served as control groups. Their diagnoses were made according to international criteria laid down in consensus statements of the International Bárány Society for Neuro-Otology (https://www.jvr-web.org/ICVD.html) and the International Headache Society (https://www.ichd-3.org) [31, 32]. As signs of central vestibular dysfunction, all patients of subgroups 1–3 showed central ocular motor dysfunctions such as reduced velocity of saccades, hyper-/hypometric saccades, gaze-evoked or rebound nystagmus, abnormal VOR suppression, and positioning downbeat nystagmus.

### Vertigo Handicap Questionnaire (VHQ)

All patients completed the VHQ, which consists of 25 well-established items to measure the self-perceived vertigo-related impairment [11]. The VHQ is scored on a 5-point Likert scale (0 = never; 4 = always), with 12 questions inverted to avoid response bias. The total score ranges from 0 to 100 (0 indicating no impairment and higher scores implying higher impairment due to vertigo or dizziness). There are two established subscales in the German version, comprising vertigo-related anxiety (VHQ-ANX; range: 0–4) and handicapped activity (VHQ-ACT; range: 0–4) [11]. A value close to 0 can be expected in healthy controls.

### Static posturography

As an objective measure of postural in-/stability, static posturography was performed in standardized manner using a stabilometer platform (Kistler 9261A, Kistler Group, Sindelfingen, Germany) for off-line analysis (sampling frequency 40 Hz) [33]. Anterior/posterior (y) and lateral (x) body sway (centre of pressure (COP) in mm), as well as body weight (z, force in Nm) measurements were collected for each of the following conditions capturing standing situations under visual and/or somatosensory disturbance: (1) standing on a hard surface with eyes open, and (2) eyes closed, (3) standing on a foam rubber slab with eyes open and (4) eyes closed. To analyse the variability in sway in a single variable for each condition, the combined root mean squared sway (RMS, in mm^2^) in x and y plane was evaluated (Supplementary Table 2).

### Statistical analysis

We report mean values and standard deviation for continuous variables and absolute and relative frequencies for categorical variables. For the comparison of the results of VHQ from central vestibular disorders and BVP as a reference group single t-tests were performed. Group differences were tested using the non-parametric Kruskal–Wallis test with Bonferroni-adjusted post-hoc analysis for differences between the diagnosis groups. To control for potential confounding and to identify factors associated with the VHQ and its subscores we applied multiple linear regression models. All models were controlled for age, sex and polyneuropathy. We report the regression coefficient together with the respective 95% confidence interval. Multicollinearity was checked using the generalized variance inflation factor [34]. A generalized variance inflation factor greater than five was considered as problematic. Postural sway variability means between patient groups were tested with a Kruskal–Wallis chi-squared test for each condition individually and p-values were corrected with a Bonferroni correction to α = 0.0125, and pairwise post-hoc comparisons were tested with Wilcoxon rank sum test with Benjamini–Hochberg adjustment of p-values. To test for a relationship between objective postural stability and VHQ subscores, pairwise correlations between the four posturography variables and the two sub-scores were calculated for the total cohort and separately for the individual diagnoses. SPSS (SPSS Statistics 27.0.1.0, IBM, 2020, Armonk/NY, USA) and R version 4.2.0/RStudio Version 2022.07.1 + 554 (with the following packages: tidyverse, readxl, car, rstatix, with ggpubr for creating figures) were used for statistical analyses.

### Protocol approval and patient consent

The data protection policies and Institutional Review Board of the Ludwig-Maximilians-Universität München, Germany, approved the study (No. 414-15) and all patients gave informed consent. The study was performed in accordance with the ethical standards laid down in the 1964 Declaration of Helsinki and its later amendments.

## Results

### Patient characteristics

The mean age of the 371 patients was 55.8 years, fifty-eight percent were female. Patients with VM differed from the rest of the cohort in age and sex distribution according to their typical clinical manifestation [35] (Table 1). Peripheral vestibular function was normal in almost all central vestibular disorders, except for CANVAS patients with expected peripheral vestibular hypofunction bilaterally [36]. Unsurprisingly, also some patients with cerebellar dysfunction showed pathologically reduced VOR gain or increased refixation saccades (32.7% in COD, 47.9% in CA), due to flocculo-vestibular impairment [37].

**Table 1:** Patient characteristics and results of VHQ questionnaire, pathological findings are indicated in bold

	COD	CA	APS	VM	BVP
N	61	63	28	154	65
Age at presentation (years)	66.9 ± 12.8 [range: 36–88]	63.6 ± 15.2 [range: 22–88]	72.9 ± 8.7 [range: 50–89]	42.8 ± 14.0 [range: 19–82]	61.2 ± 19.6 [range: 20–93]
Sex (female | male)	54% | 46%	52% | 48%	43% | 57%	72% | 28%	38% | 62%
Neuro-otological characteristics					
VOR gain [°/s]	0.82 ± 0.20 [range: 0.30–1.27]	**0.66** ± 0.28 [range: 0.03–1.25]	0.92 ± 0.22 [range: 0.52–1.49]	0.93 ± 0.10 [range: 0.60–1.15]	**0.34** ± 0.21 [range: 0.0–0.89]
Bithermal caloric response [°/s]	32.3 ± 13.7 [range: 9.0–71.4]	30.0 ± 27.6 [range: 2.0–128.4]	25.1 ± 11.8 [range: 7.0–54.5]	24.4 ± 12.4 [range: 8.0–65.9]	**3.6** ± 2.3 [range: 0.0–10.5]
SVV [°]	− 0.6 ± 1.7 [range: -5.0–3.2]	0.3 ± 2.1 [range: -4.4–7.0]	− 0.5 ± 2.1 [range: -6.0–5.9]	0.0 ± 1.0 [range: -8.0–4.0]	− 0.4 ± 1.9 [range: -5.0–4.0]
VHQ total score	47.7 ± 17.4 [range: 13–85]	55.5 ± 17.3 [range: 10–84]	56.1 ± 15.6 [range: 26–85]	48.9 ± 16.7 [range: 13–93]	48.4 ± 15.6 [range: 13–81]
VHQ-ACT	1.96 ± 0.76 [range: 0.33–3.33]	2.29 ± 0.68 [range: 0.54–3.47]	2.34 ± 0.59 [range: 1.00–3.53]	1.82 ± 0.69 [range: 0.27–3.80]	1.99 ± 0.70 [range: 0.40–3.20]
VHQ-ANX	2.07 ± 0.90 [range: 0.40–3.83]	2.41 ± 0.74 [range: 0.33–4.00]	2.35 ± 0.87 [range: 0.67–4.00]	2.50 ± 0.87 [range: 0.50–4.00]	2.16 ± 0.89 [range: 0.60–3.50]

VOR gain is given as mean gain of both sides (left and right) in [°/s], caloric response as mean slow phase velocity of the bithermal induced nystagmus [°/s]. Results of the Vertigo Handicap Questionnaire (VHQ) total score as well of the subscores for handicapped activity (VHQ-ACT) and anxiety (VHQ-ANX) for the different central vestibular disorders and BVP

*VOR* vestibulo-ocular reflex, *SVV* subjective visual vertical, *BVP* bilateral vestibulopathy, *COD* cerebellar ocular motor disorders, *CA* cerebellar ataxia, *APS* atypical parkinsonian syndromes, *VM* vestibular migraine

**Fig. 1:**
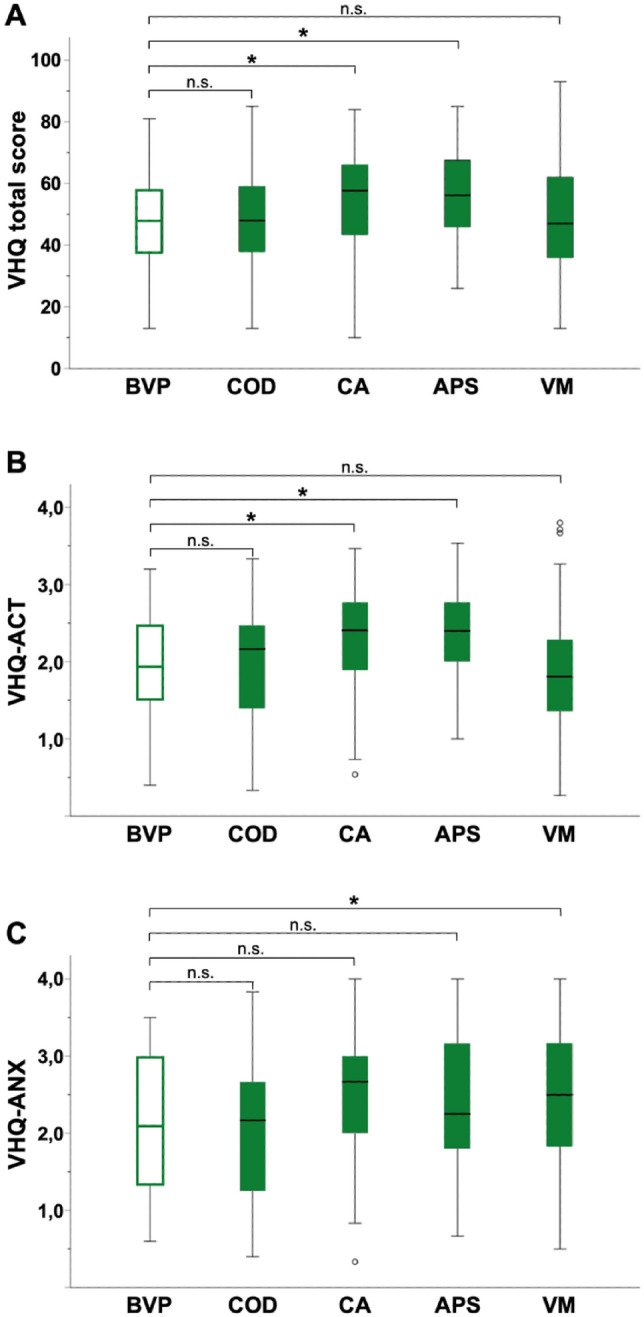
Results of the Vertigo Handicap Questionnaire (VHQ) given as (**A**) total score as well as its subscores for (**B**) handicapped activity (VHQ-ACT) and (**C**) vestibular related anxiety (VHQ-ANX) for the whole patient collective. t-test analyses assessing statistical differences between central vestibular disorders and the peripheral control group BVP revealed significant differences for the mean values of the VHQ total score and VHQ-ACT for CA and APS. BVP, COD and VM showed comparable VHQ total score and VHQ-ACT scores without significant difference (n.s.). The VHQ-ANX subscore differed significantly between BVP and VM. *p values indicate statistical significance (p < 0.05). *BVP* bilateral vestibulopathy, *COD* cerebellar ocular motor disorders, *CA* cerebellar ataxia, *APS* atypical parkinsonian syndromes, *VM* vestibular migraine

The VHQ score ranged from 10 to 93 (mean 50.27 ± 16.79) and was lowest in COD and highest in APS (Table 1). CA and APS patients showed significantly higher VHQ scores than the peripheral control group BVP (p = 0.015 and p = 0.031, see Fig. 1). The VHQ-ACT was lowest in VM and highest in APS. Again, significant differences compared to BVP were seen in CA (p = 0.014) and APS (p = 0.022) patients. The VHQ-ANX was lowest in COD and highest in VM. Significant differences were found for VM compared to BVP (p = 0.015), whereas the other central vestibular disorders showed no statistical significance (all p > 0.05) (Fig. 1).

A multivariate linear regression model that adjusted for age, sex and polyneuropathy confirmed significant differences for the distinct diagnoses for the VHQ (p = 0.0388), the VHQ-ACT (p = 0.0021), and the VHQ-ANX (p = 0.0008) (Table 2). Subgroup analyses revealed significant differences of the VHQ total score and VHQ-ACT only for CA (VHQ total score p = 0.0376; VHQ-ACT p = 0.0241). The significant difference between BVP and VM (p = 0.0057) in the VHQ-ANX was verified. In addition, age, polyneuropathy and being female, all significantly increased the VHQ-ANX (all p < 0.05). We performed a separate regression model for VHQ-ANX for each patient subgroup to investigate if the association remained stable in each strata (Table 3). A significant effect of polyneuropathy was seen in the BVP and the COD group, but not in the three other groups. Age was significantly associated with anxiety only in the VM group showing higher VHQ values with increasing age. Pathology in vestibular examination did not correlate with VHQ in a multivariate linear regression analysis (Supplement Table 3).

**Table 2:** Multivariate linear regression analysis for VHQ total score, VHQ-ACT, and VHQ-ANX: for the complete study population of 371 patients

	VHQ total score			VHQ-ACT			VHQ-ANX		
	β	95% CI	p	β	95% CI	p	β	95% CI	p
Diagnosis	–	–	0.0338*	–	–	0.0021*	–	–	0.0008*
COD	− 1.93	[− 7.83; 3.97]		− 0.06	[− 0.31; 0.19]		− 0.23	[− 0.54; 0.08]	
CA	6.18	[0.36; 12.01]		0.28	[0.04; 0.53]		0.13	[− 0.18; 0.45]	
APS	6.44	[− 1.04; 13.91]		0.30	[− 0.01; 0.62]		0.05	[− 0.35; 0.45]	
VM	2.07	[− 3.31; 7.45]		− 0.13	[− 0.36; 0.10]		0.40	[0.12; 0.69]	
Age	0.009	[− 0.02; 0.21]	0.1210	0.00	[0.00; 0.01]	0.1399	0.001	[0.00; 0.01]	0.0193*
Male sex	− 1.93	[− 5.49; 1.63]	0.2864	− 0.09	[− 0.24; 0.06]	0.2519	− 0.32	[− 0.50; − 0.13]	0.001*
PNP	3.08	[− 2.06; 8.22]	0.2401	0.00	[− 0.22; 0.22]	0.9886	0.31	[0.04; 0.58]	0.0245*

Regression coefficients (β), 95% confidence interval as well as p-values are given. Statistically significant values are indicated by *

*COD* cerebellar ocular motor disorders, *CA* cerebellar ataxia, *APS* atypical parkinsonian syndromes, *VM* vestibular migraine, *PNP* polyneuropathy

**Table 3:** Multivariate linear regression of variables age, sex and polyneuropathy (PNP) for VHQ anxiety (VHQ ANX) for the different diagnoses

	VHQ-ANX		
	β	95% CI	p
BVP			
Age	− 0.01	[− 0.02; 0.01]	0.3308
Male sex	− 0.32	[− 0.8; 0.16]	0.1884
PNP	0.8	[0.19; 1.41]	0.0108*
COD			
Age	0	[− 0.01; 0.02]	0.6487
Male sex	− 0.28	[− 0.75; 0.19]	0.2407
PNP	0.56	[0.02; 1.09]	0.043*
CA			
Age	0.01	[− 0.01; 0.02]	0.3394
Male sex	− 0.36	[− 0.76; 0.04]	0.074
PNP	0.04	[− 0.39; 0.46]	0.8597
APS			
Age	0.01	[− 0.01; 0.02]	0.3394
Male sex	− 0.51	[− 1.31; 0.28]	0.1959
PNP	− 0.04	[− 1.02; 0.93]	0.9246
VM			
Age	0.02	[0.01; 0.03]	0.0022*
Male sex	− 0.29	[− 0.60; 0.02]	0.0635
PNP	− 0.27	[− 2.00; 1.46]	0.7579

Statistically significant p values are indicated by *

*BVP* bilateral vestibulopathy, *COD* cerebellar ocular motor disorders, *CA* cerebellar ataxia, *APS* atypical parkinsonian syndromes, *VM* vestibular migraine, *PNP* polyneuropathy

Group comparisons within the central vestibular disorders revealed significant differences for VHQ total score and its subscores (VHQ total score p = 0.037; VHQ-ACT p < 0.001; VHQ-ANX p = 0.005). Post-hoc Bonferroni analysis showed statistically significant differences between the different diagnoses (Table 4): VHQ total score differed significantly between VM and CA (p = 0.021), whereas VHQ-ACT differed between VM and CA (p < 0.001) as well as between VM and APS (p = 0.001). Significant differences in VHQ-ANX were only evident between VM and COD (p = 0.023).

**Table 4:** Differences between the central vestibular disorders: Kruskal–Wallis one-way analysis of variance for VHQ and subscores were performed

	VHQ sum x^2^(3) = 8.484, p = 0.037				VHQ activity x^2^(3) = 29.267, p = < 0.001				VHQ anxiety x^2^(3) = 12.802, p = 0.005			
	VM	COD	CA	APS	VM	COD	CA	APS	VM	COD	CA	APS
VM	–				–				–			
COD	1.000	–			0.559	–			0.023*	–		
CA	0.021*	0.056	–		< 0.001*	0.095	–		1.000	0.303	–	
APS	0.191	0.235	1.000	–	0.001*	0.154	1.000	–	1.000	1.000	1.000	**–**

Shown are the p-values of the Bonferroni post-hoc analysis to test for statistical significance between groups. *p-values were considered statistically significant

*BVP* bilateral vestibulopathy, *COD* cerebellar ocular motor disorders, *CA* cerebellar ataxia, *APS* atypical parkinsonian syndromes, *VM* vestibular migraine

### Posturographic measurements

**Fig. 2:**
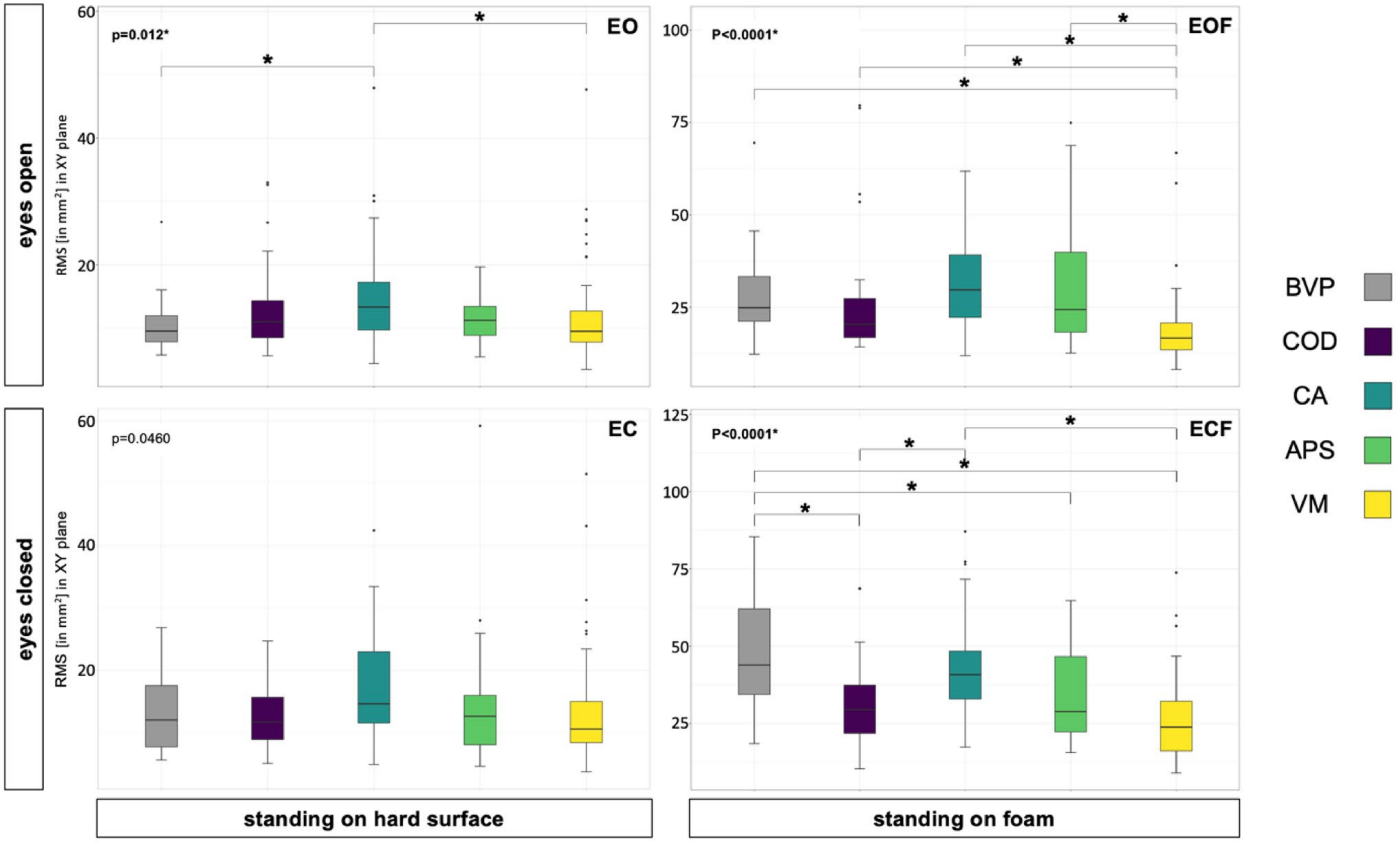
Postural sway variability data for 192 patients (BVP = 28, COD = 36, CA = 39, APS = 21, VM = 68) for condition eyes open on a hard surface (EO), eyes closed on a hard surface (EC) standing on foam with eyes open (EOF), standing on foam with eyes closed (ECF). *p values indicate statistical significance (p < 0.05). *RMS* root mean squared sway, *BVP* bilateral vestibulopathy, *COD* cerebellar ocular motor disorders, *CA* cerebellar ataxia, *APS* atypical parkinsonian syndromes, *VM* vestibular migraine

Three of the four posturographic conditions showed significant differences in RMS between patient groups (EO: p = 0.01195; EC: p = 0.0463; EOF: p < 0.001, ECF: p < 0.001; significance was corrected to p = 0.0125). Posthoc pairwise comparisons revealed that under normal standing conditions with eyes open (EO) CA had highest sway variability, significantly higher than BVP or VM (adjusted p-values p = 0.041 and p = 0.016 respectively). With increasing demands of standing performance, eyes open on foam (EOF), VM patients showed significantly less variability of sway than all other patient groups (adjusted p-values: VM-BVP p < 0.001, VM-COD p = 0.001, VM-CA p < 0.001, VM-APS p = 0.003). For the condition eyes closed on foam (ECF), BVP patients showed the highest sway variability, significantly higher than COD, APS and VM patients; CA patients had higher sway variability than COD and VM (adjusted p-values: BVP-COD p < 0.001, BVP-APS p = 0.044, BVP-VM p < 0.001, CA-COD p = 0.005, CA-VM p < 0.001) (Fig. 2).

**Fig. 3:**
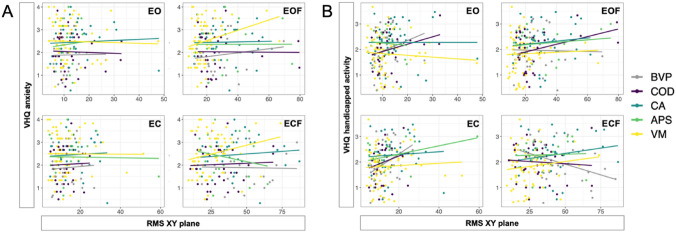
Correlation analysis of posturographic sway variability (RMS) and subjective scoring in anxiety (VHQ-ANX) (**A**) and handicapped activity (VHQ-ACT) (**B**) for 192 patients for condition eyes open on a hard surface (EO), eyes closed on a hard surface (EC) standing on foam with eyes open (EOF), standing on foam with eyes closed (ECF). A significant correlation across all patients was seen for VHQ-ACT/EC (r = 0.1950; p = 0.0072) and VHQ-ACT/EOF (r = 0.1972; p = 0.0065). *RMS* root mean squared sway, *BVP* bilateral vestibulopathy, *COD* cerebellar ocular motor disorders, *CA* cerebellar ataxia, *APS* atypical parkinsonian syndromes, *VM* vestibular migraine

For the total patient cohort, root mean squared sway (RMS) was not correlated with anxiety, VHQ-ANX (EO: p = 0.913; EC: p = 0.381; EOF: p = 0.535; ECF: p = 0.966) (Fig. 3A). However, sway variability was correlated with handicapped activity, VHQ-ACT, for the conditions eyes closed (EC, p = 0.0072) and eyes open on foam (EOF, p = 0.0065) but not eyes open (EO, p = 0.0578) or eyes closed on foam (ECF, p = 0.293) (Fig. 3B). Separating out the individual patient groups showed that these correlations vary across groups (Fig. 3), but subgroup analyses did not reveal any significant correlations.

## Discussion

The main findings of our study on subjective vertigo-related physical and psychological impairment based on the Vertigo Handicap Questionnaire (VHQ) in relation to objective stance function measured by posturography in different types of central vestibular dysfunction were as follows:

1. The VHQ, which assesses impairment in daily life due to vertigo or dizziness, was generally increased in central vestibular disorders, even if compared to peripheral vestibular disorders, but showed marked differences depending on the distinct diagnoses.
2. Patients with VM showed the lowest vestibular-related activity impairment. Patients with COD and BVP reported similarly low levels, although BVP patients suffer from relevant postural deficits. As expected, CA and APS reported significantly higher handicapped activity compared to BVP and VM.
3. Patients with chronic central vestibular disorders reported vertigo-related anxiety, VRA, at comparably low levels as BVP patients. Only patients with VM showed significantly higher anxiety scores compared to the other central vestibular disorders.
4. The multivariate linear regression analyses revealed an overall effect of age, sex and peripheral sensory impairment (polyneuropathy) on VRA. The subgroup analyses revealed that polyneuropathy increased the anxiety in BVP and COD, but not in the other groups. VM was the only subgroup with a positive correlation between anxiety and ageing. Gender had no effect on VRA in the subgroup analyses.
5. Postural instability increased with higher demands of the stance conditions. Patient subgroups differed in their postural instability, and CA showed consistently higher instability than other patient groups, whereas VM showed consistently low sway variability over all conditions.
6. Objective sway variability correlated with subjective activity impairment but did not correlate with subjective anxiety, VRA. This was especially true for VM, who showed the lowest sway variability and activity impairment, but the highest anxiety scores.

The starting point of our study was the previous finding that patients with a loss of peripheral vestibular function, such as unilateral (UVP) or bilateral vestibulopathy (BVP) show relatively low anxiety levels compared to other vestibular disorders in a specialised vertigo centre [12]. This led to the hypothesis that an intact peripheral vestibular system is a prerequisite for the development of VRA [15]. While UVP is a monophasic event that rarely causes ongoing disabilities after complete central compensation [38], BVP leads to a persistent vestibular and postural impairment associated with an increased risk of recurrent and injurious falls [13]. Surprisingly, the latter patients have only low to moderate fear of falls [13]. Accordingly, BVP patients do not have an increased susceptibility to fear of heights, indicating a generally lowered perception of vestibular related anxiety in patients with BVP [39].

Numerous connections at lower and higher brain levels exist between the vestibular and emotional systems, including various central areas concerned with anxiety perception, e.g., projections to the parabrachial nucleus and its reciprocal connections with amygdala, infralimbic and insular cortex, and hypothalamus [16, 20, 40]. These connections indicate a close structural and functional connection between vestibular input and emotional processing, which is reflected in the rates of vestibular and affective comorbidities [6]. However, the complex interplay between these two systems, in particular the role of central vestibular impairment in the development of VRA remains largely unknown.

In our study patients with chronic central vestibular disorders surprisingly reported comparably low levels of anxiety as patients with BVP and without significant differences within these patient groups. Even patients with CA, who demonstrated the highest subjective activity impairment and stance instability in posturography, showed relatively low VRA. Overall, postural instability correlated with self-reported activity impairment across all diagnoses, but not with VRA. This suggests that, in line with previous studies [41, 42], VRA is not related to an objective physical impairment of stance and gait regulation. Rather, the proper function of the central as well as the peripheral vestibular system is relevant for the development of vertigo-related anxiety.

Compared to earlier data, it is important to note that Lahmann and co-workers reported manifest psychiatric comorbidity in dizzy patients [6], whereas we evaluated specific vertigo-related anxiety in patients with vestibular disorders that do not fulfil the criteria for an associated psychiatric disorder. However, the results of the two studies fit nicely together, since manifest anxiety disorders were even rare comorbidities in their inhomogeneous group of central vertigo (18.4%) and BVP patients (17.8%) compared to the total patients’ average (28.9%) [6].

Our study provides new insights into different subgroups of central vestibular disorders:

Increasing evidence indicates the cerebellum’s influential role in various higher cognitive and emotional processes, particularly for susceptibility to anxiety [21, 43, 44]. In his internal model hypothesis, Hilber proposed that cerebellar or vestibular disorders induce an alteration of sensory information by altering the integration process of exteroceptive and proprioceptive information [20]. This leads to false anticipation as well as motor and coordinative and subsequently also social interaction with the environment, resulting in stress and anxiety. In fact, Schlick and co-workers found an association between high levels of recurrent falls and high levels of specific fear of falling in cerebellar disorders [13]. In contrast, in our study we did not find a relationship between the VRA and postural instability in patients with cerebellar disorders, not even in the considerably more activity-handicapped subgroup of CA.

Already at an early stage neurodegenerative disorders are often associated with balance disorders and spatial disorientation, assumed to result from a disturbed central processing of multisensory information with involvement of the vestibular, visual, somatosensory and motor systems [45]. An autopsy-confirmed case series found that dizziness was indeed the first clinical symptom in 4.2 to 7.7% of the subgroup of patients with atypical Parkinson's syndromes (APS) [46], who develop high postural instability with increased risk of falls during the course of the disease [13]. In our study, however, VRA levels in APS were similar to those in BVP, although the subjective handicapped activity and objective postural instability of these patients were high. This further points towards a more complex interplay between vestibular function and the development of specific VRA.

VM patients stood out from the other central vestibular disorders showing an inverse pattern of high VRA despite low subjective handicapped activity and good postural performance. This is consistent with a prospective one-year follow-up study that reported ongoing high psychological distress with significantly elevated “vertigo-induced anxiety” levels in VM [5]. A systematic review confirmed an overall strong and continuing positive relationship between migraine and anxiety [47]. Potential pathophysiological mechanisms include altered signalling mechanisms in the brainstem and thalamus as well as trigeminovascular activation [47] and disturbances in intracerebral GABAergic inhibition as in panic disorders [5]. Notably, VM is the only central vestibular disorder of episodic type addressed in our study with vertigo attacks that typically do not cause significant vestibular deficits in the interictal interval. The acute and unpredictable occurrence of VM attacks could be a trigger for enhanced VRA [3].

The majority of our patients, with the exception of VM patients, were above 60 years of age. Age is a relevant factor for dizziness and balance disorders due to increasing comorbidity [48]. No overall impact of age, sex or sensory impairment (polyneuropathy) was found on vertigo-related handicap and activity impairment. However, a significantly increased VRA was evident in the presence of polyneuropathy in BVP and COD. It is well-known that proprioceptive and visual feedback is of particular relevance for BVP, which is reflected by posturography under visual and sensory perturbation [14]. Notably, only the subgroup of VM showed an increase of VRA with higher age, although the incidence of VM typically decreases with advanced age and persistent relevant vestibular or ocular motor dysfunction in the attack-free interval is rare [49, 50]. This further points towards the special entity of VM and its interplay with the anxiety system.

In summary, subjective and objective vestibular-related impairment is not necessarily correlated with vestibular-related anxiety in central vestibular disorders. Rather, our findings support the view, that in addition to an intact peripheral system, also an intact central vestibular system might also serve as a prerequisite for the development specific VRA.

The main limitation of our study was its retrospective approach with a potential selection bias due to referral to a tertiary vertigo centre. Consequently, patients included might be more severely burdened, and the data cannot be easily transferred to the general population. Retrospectively, we were not able to perform a wider range of standardised psychiatric questionnaires, but a careful evaluation of current complaints and patient history including psychiatric comorbidities was routinely performed during the presentation. Further studies are needed to investigate the complex interplay between the vestibular system, anxiety, vestibular-related anxiety, and psychiatric comorbidities as well as their influences on daily living and quality of life in more detail.

### Supplementary Information

Below is the link to the electronic supplementary material.

Supplementary file1 (DOCX 19 KB)

## Data Availability

All data supporting the findings of this study are provided anonymously within the paper and its Supplementary Information. Further data are available on reasonable request from the corresponding author.
