# Supplementary material for: Anxiety and physical impairment in patients with central vestibular disorders
Source: J Neurol. 2023 Aug 8;270(11):5589–99. doi: 10.1007/s00415-023-11871-3 (PMC10576724; doi:10.1007/s00415-023-11871-3)
Supplement: Supplementary file 1 — Supplementary file1 (DOCX 19 KB) [file 415_2023_11871_MOESM1_ESM.docx]

**Supplement**

Supplement - Table 1:

| **Vestibular function –**  **all central vestibular disorders** | | | **vHIT** | | **sum** |
| --- | --- | --- | --- | --- | --- |
|  |  |  | **normal vestibular function** | **bilateral vestibular hypofunction** |  |
| **bithermal caloric irrigation** | **normal vestibular function** | | **81,3%** | **15,7%** | **97,0%** |
|  | **bilateral vestibular hypofunction** | | **0,7%** | **2,2%** | **3,0%** |
| **sum** | | | **82,1%** | **17,9%** |  |
|  | | | | | |
| **VM** | | | vHIT | | sum |
|  |  |  | normal vestibular function | bilateral vestibular hypofunction |  |
| bithermal caloric irrigation | | normal vestibular function | **95,1%** | **4,2%** | **99,3%** |
|  |  | bilateral vestibular hypofunction | **0,7%** | **0,0%** | **0,7%** |
| sum | | | **95,8%** | **4,2%** |  |
|  | | | | | |
| **COD** | | | vHIT | | sum |
|  |  |  | normal vestibular function | bilateral vestibular hypofunction |  |
| bithermal caloric irrigation | | normal vestibular function | 67,3% | 32,7% | 100,0% |
|  |  | bilateral vestibular hypofunction | 0,0% | 0,0% | 0,0% |
| sum | | | **67,3%** | **32,7%** |  |
|  | | | | | |
| **CA** | | | vHIT | | sum |
|  |  |  | normal vestibular function | bilateral vestibular hypofunction |  |
| bithermal caloric irrigation | | normal vestibular function | **50,0%** | **35,4%** | **85,4%** |
|  |  | bilateral vestibular hypofunction | **2,1%** | **12,5%** | **14,6%** |
| sum | | | **52,1%** | **47,9%** |  |
|  | | | | | |
| **APS** | | | vHIT | | sum |
|  |  |  | normal vestibular function | bilateral vestibular hypofunction |  |
| bithermal caloric irrigation | | normal vestibular function | **91,7%** | **8,3%** | **100,0%** |
|  |  | bilateral vestibular hypofunction | **0,0%** | **0,0%** | **0,0%** |
| sum | | | **91,7%** | **8,3%** |  |

Supplement - Table 1: results from vestibular diagnostic in patients with central vestibular disorders. Results were classified by bithermal caloric irrigation (row; normal vestibular function: mean gain of bithermal irrigation per side ≥ 6 °/s) and vHIT (column; normal vestibular function: mean gain ≥ 0,7 °/s). Abbreviations: BVP = bilateral vestibulopathy; COD = cerebellar ocular motor disorders; CA = cerebellar ataxia; APS = atypical parkinsonian syndromes; VM = vestibular migraine. vHIT = video head impulse test.

Supplement - Table 2:

| **RMS**  [in mm^2^] | **EO** | **EC** | **EOF** | **ECF** |
| --- | --- | --- | --- | --- |
| **COD** | 12.96 ± 6.74 | 12.73 ± 5.20 | 26.35 ± 15.89 | 30.57 ± 13.88 |
| **CA** | 15.01 ± 8.22 | 17.29 ± 8.69 | 31.57 ± 13.01 | 42.15 ± 16.40 |
| **APS** | 11.61 ± 4.14 | 15.28 ± 12.05 | 30.07 ± 16.99 | 33.74 ± 14.83 |
| **VM** | 11.52 ± 7.14 | 13.15 ± 8.54 | 18.68 ± 9.61 | 25.56 ± 12.05 |
| **BVP** | 10.60 ± 4.37 | 13.22 ± 6.11 | 27.92 ± 11.84 | 46.98 ± 17.54 |

Supplement - Table 2: Results of posturographic sway variability (sum of RMS in XY-plane) for the different central vestibular disorders and BVP. Abbreviations: RMS = root mean squared sway; BVP = bilateral vestibulopathy; COD = cerebellar ocular motor disorders; CA = cerebellar ataxia; APS = atypical parkinsonian syndromes; VM = vestibular migraine.

Supplement - Table 3:

|  | | **VHQ total score** | | | **VHQ-ACT** | | | **VHQ-ANX** | | |
| --- | --- | --- | --- | --- | --- | --- | --- | --- | --- | --- |
|  |  | β | 95% CI | p | β | 95% CI | p | β | 95% CI | p |
| **Neuro-otological characteristics** | VOR gain [°/s] | 4.43 | [-4.11; 12.96] | 0.3081 | 0.05 | [-0.32; 0.41] | 0.795 | 0.14 | [-0.32; 0.6] | 0.546 |
|  | bithermal caloric response [°/s] | -0.03 | [-0.17; 0.11] | 0.6823 | 0 | [-0.01; 0.01] | 0.9601 | 0 | [-0.01; 0.01] | 0.9877 |
|  | SVV [°] | 0.18 | [-1.13; 1.5] | 0.782 | -0.02 | [-0.08; 0.04] | 0.5128 | 0.02 | [-0.05; 0.08] | 0.6605 |

Supplement Table 3: Multivariate linear regression analysis of the effect of neuro-otological results on VHQ total score, VHQ-ACT, and VHQ-ANX for the complete study population of 371 patients. Regression coefficients (ß), 95% confidence interval as well as p-values are given. No statistically significant effect was seen. Abbreviations: VHQ = Vertigo Handicap Questionnaire; VHQ-ACT = VHQ subscale for handicapped activity; VHQ-ANX = VHQ subscale for anxiety; VOR = vestibulo-ocular reflex; SVV = subjective visual vertical.
